# Safe standard remnant liver volume after hepatectomy in HCC patients in different stages of hepatic fibrosis

**DOI:** 10.1186/s12893-021-01065-x

**Published:** 2021-01-23

**Authors:** Zhiming Zhang, Gaoxiong Ouyang, Peng Wang, Yuan Ren, Yukai Liu, Jun Chen, Yumei Zhang, Jianyong Liu, Lequn Li

**Affiliations:** 1 Department of Hepatobiliary Surgery, Guangxi Medical University Cancer Hospital, No. 71 Hedi Road, Nanning, 530021 Guangxi Zhuang Autonomous Region China; 2 Department of Radiology, Guangxi Medical University Cancer Hospital, Nanning, 530021 Guangxi Zhuang Autonomous Region China; 3 Department of Pathology, Guangxi Medical University Cancer Hospital, Nanning, 530021 Guangxi Zhuang Autonomous Region China; 4 Department of Chemotherapy, Guangxi Medical University Cancer Hospital, Nanning, 530021 Guangxi Zhuang Autonomous Region China

**Keywords:** Standard remnant liver volume, Hepatectomy, HCC, Hepatic fibrosis, Cirrhosis

## Abstract

**Background:**

To determine the standard remnant liver volume (SRLV) threshold to avoid postoperative hepatic insufficiency inpatients in different stages of hepatic fibrosis who undergo right hemi-hepatectomy.

**Methods:**

Data for 85 patients at our single medical center were analysed prospectively to examine whether the following factors differed significantly between those who experienced postoperative hepatic insufficiency and those who did not: height, prothrombin time, remnant liver volume, SRLV or hepatic fibrosis stage.

**Results:**

Logistic regression showed SRLV and hepatic fibrosis stage to be independent risk factors for postoperative hepatic insufficiency. The threshold SRLV for predicting insufficiency was 203.2 ml/m^2^ across all patients [area under receiver operating characteristic curve (AUC) 0.778, sensitivity 66.67%, specificity 83.64%, p<0.0001), 193.8 ml/m^2^ for patients with severe hepatic fibrosis (AUC 0.938, sensitivity 91.30%, specificity 85.71%, p<0.0001), and 224.3 ml/m^2^ for patients with cirrhosis (AUC 0.888, sensitivity 100%, specificity 64.29%, p<0.0001).

**Conclusions:**

Right hemi-hepatectomy may be safer in Chinese patients when the standard remnant liver volume is more than 203.2 ml/m^2^ in the absence of hepatic fibrosis or cirrhosis, 193.8 ml/m^2^ in the presence of severe hepatic fibrosis or 224.3 ml/m^2^ in the presence of cirrhosis.

## Background

Hepatocellular carcinoma (HCC) is the third leading cause of cancer-related mortality and accounts for one-fifth of all cancer cases worldwide [1]. Numerous treatments are in use, including surgical resection, transcatheter arterial chemoembolisation, radiofrequency ablation, radiotherapy, chemotherapy, and immunotherapy [2, 3]. Liver resection remains the best treatment for HCC patients who cannot undergo liver transplantation [4]. However, sufficient remnant liver volume must be retained to meet the demands of metabolism and maintain homeostasis. Otherwise, the patient may suffer impaired liver function, liver failure and death [5, 6]. Therefore, it is particularly important to determine how much remnant liver volume should be retained after hepatectomy in each patient.

Standard remnant liver volume (SRLV), in which remnant liver volume is measured using multi-slice spiral computer tomography (CT) and then normalised to body surface area, is the most practical and accurate way to assess liver reserve function and predict postoperative hepatic insufficiency [7]. While one study reported that SRLV < 250 mL / m^2^ is associated with significantly higher risk of hepatic insufficiency than higher SRLV [8], other studies have reported different thresholds [7]. Therefore there is no consensus on an SRLV threshold for postoperative hepatic insufficiency. Determining such a threshold may contribute to patient safety, especially in right hemi-hepatectomy, since the right half of the liver accounts for about 60–75% of the total liver volume [9], meaning greater risk that tissue removal will leave behind insufficient remnant liver.

The present study aimed to determine threshold SRLVs for HCC patients undergoing right hemi-hepatectomy. Since HCC patients undergoing hepatectomy in China often present with hepatic fibrosis or cirrhosis [10], we wanted to determine threshold SRLVs for these patient subgroups as well, since both conditions can strongly affect preoperative liver function and regeneration of postoperative remnant liver.

## Methods

### Ethics statement

This study was conducted in accordance with the Declaration of Helsinki and was approved by the Ethics Committee of Guangxi Medical University Cancer Hospital (approval LW2019052). All participants provided written informed consent for the use of their clinical records.

### Patient enrollment

This report is an interim analysis of an on-going prospective study involving patients who were scheduled for right hemi-hepatectomy in the Department of Hepatobiliary Surgery at the Affiliated Cancer Hospital of Guangxi Medical University. Eighty-five HCC patients were consecutively enrolled from March 2014 to February 2017. Patients matching the following criteria were enrolled: (1) right-half liver resection; (2) single or multiple tumours confined to the right hepatic lobe, which was confirmed as HCC based on postoperative pathology; (3) all liver tomography and enhancement tests were performed within one week before hepatectomy; and (4) complete pre-, intra- and postoperative clinical data.

Patients were excluded from the study if they (1) had preoperative biliary obstruction or hepatic portal cholangiocarcinoma; (2) had another malignancy such as bile duct cell carcinoma or metastatic carcinoma; (3) received preoperative cancer treatment such as radio- or chemotherapy or transcatheter arterial chemotherapy; (4) previously underwent hepatectomy; or (5) had diabetes, human immunodeficiency virus infection, or other severe diseases.

### Clinical and laboratory examinations

All baseline data, including on demographics, were collected from all patients before hepatic resection. All patients were examined using an 128-slice spiral CT (General Electric, Boston, MA, USA) at 1 week before and 1 week after liver resection. The following tests were also performed in each patient: standard liver and renal function (total bilirubin, albumin, aspartate aminotransferase, alanine transaminase), coagulation function (prothrombin time [PT]), haematological examination (white blood cells, red blood cells, platelets, haemoglobin levels), hepatitis B virus screening (HBsAg, HBsAb, HBeAg, HBeAb, HBcAb), and assay of the tumour marker alpha-foetoprotein (AFP).

### Three-dimensional liver reconstruction

**Fig. 1:**
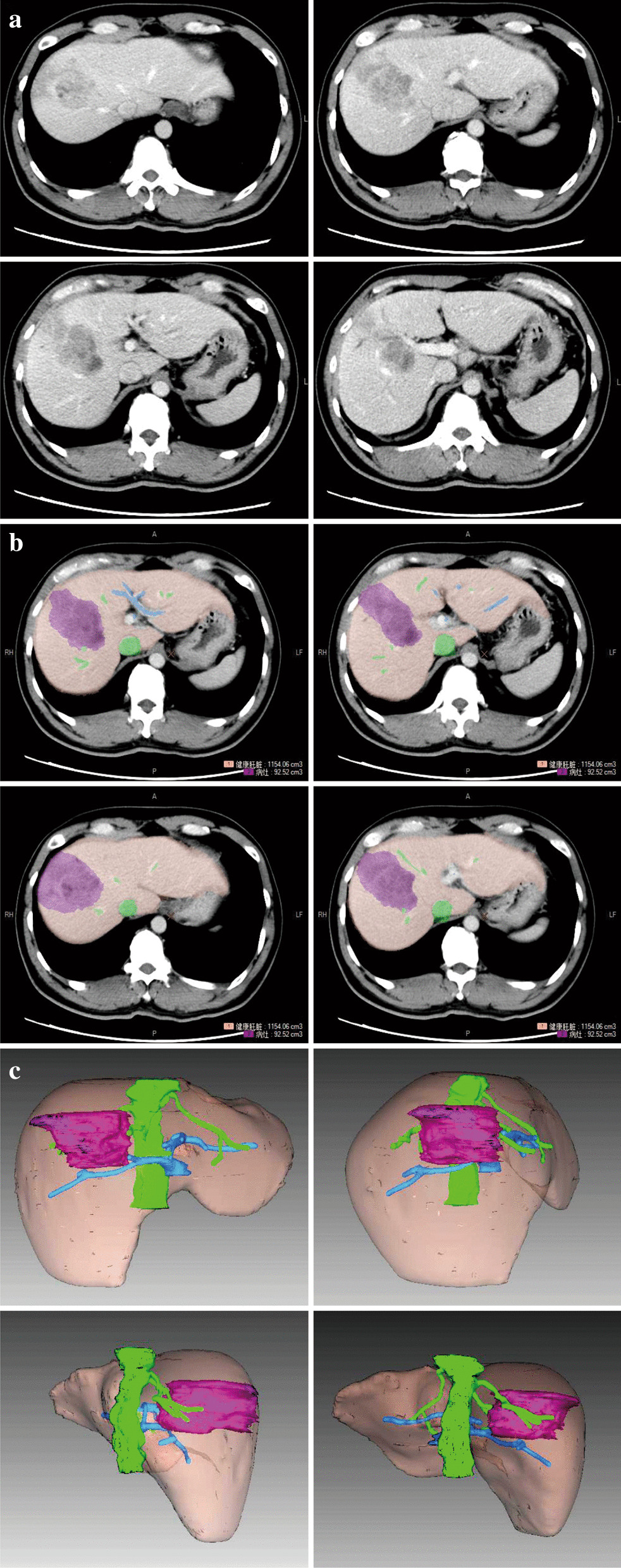
Representative micrographs showing **a** portal venous phase; **b** contours of liver, tumour, hepatic vein and portal vein depicted with different colours; and **c** 3D reconstruction of liver, tumour, hepatic vein and portal vein

Preoperative hepatectomy simulation was performed by two experienced radiologists. Original thin-slice (5 mm) CT scans, including the contiguous artery phase, portal venous phase, and delayed phase, were imported into a three-dimensional surgical simulation operation system (Myrian XP Liver 1.30.79.4, Intrasense, Montpellier, France). Portal venous phase images were used for image analysis. Different colors were assigned to different tissues, including liver, tumour, hepatic vein, and portal vein (Fig. 1a–c). Three-dimensional reconstruction was performed using an automated algorithm based on the outline of the tissue. Simulated volumes of liver, tumour, and blood vessels were also calculated.

### Simulated hepatic resection

**Fig. 2:**
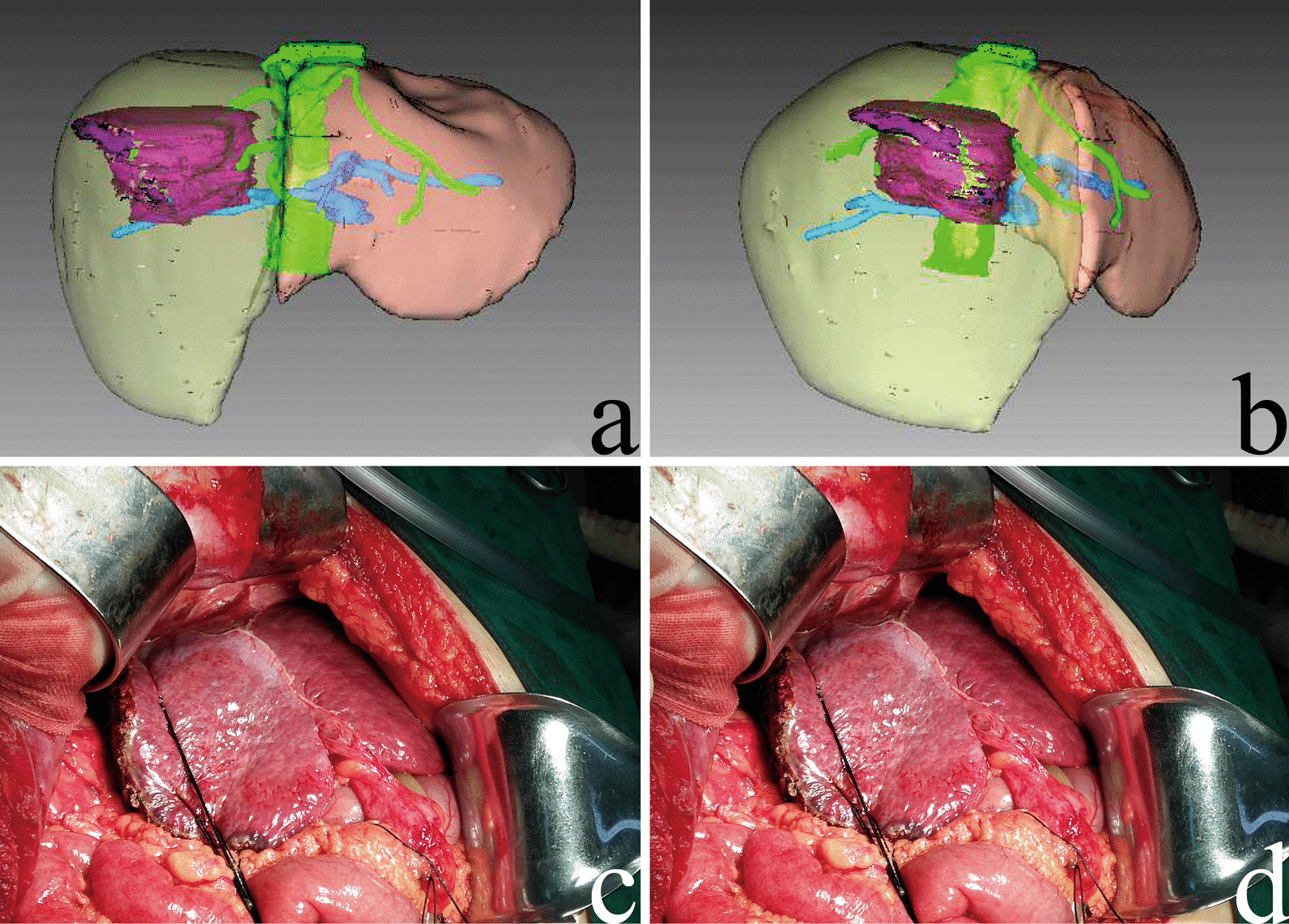
**a**, **b** Representative images of 3D simulations of right hemi-hepatectomy. **c**, **d** Intraoperative photographs showing right hemi-hepatectomy in HCC patients. Yellow area, liver volume removed; purple area, tumour volume; brown area, remnant liver volume

Simulated hepatic resection was carried out using the Myrian XP Liver system on a surgical plane generated by a 3D model in the middle hepatic vein region. Adjustments were carried out according to 2D images. Resected liver volume and remnant liver volume were calculated by the system (Fig. 2a-b). SRLV was calculated as remnant liver volume divided by body surface area [11].

### Postoperative care and diagnosis of hepatic insufficiency

All patients received the same postoperative care delivered by the same team of surgeons in the intensive care unit during the early postoperative period. Parenteral nutritional support was provided for patients with cirrhosis. Early enteric nutrition was encouraged once bowel activity resumed. Tests of liver and kidney function, coagulation function, and blood cell counts were performed on postoperative days 1, 3, 5, and 7. Postoperative hepatic insufficiency was diagnosed according to the criteria of the International Study Group of Liver Surgery (ISGLS) [12].

### Histological assessment

**Fig. 3:**
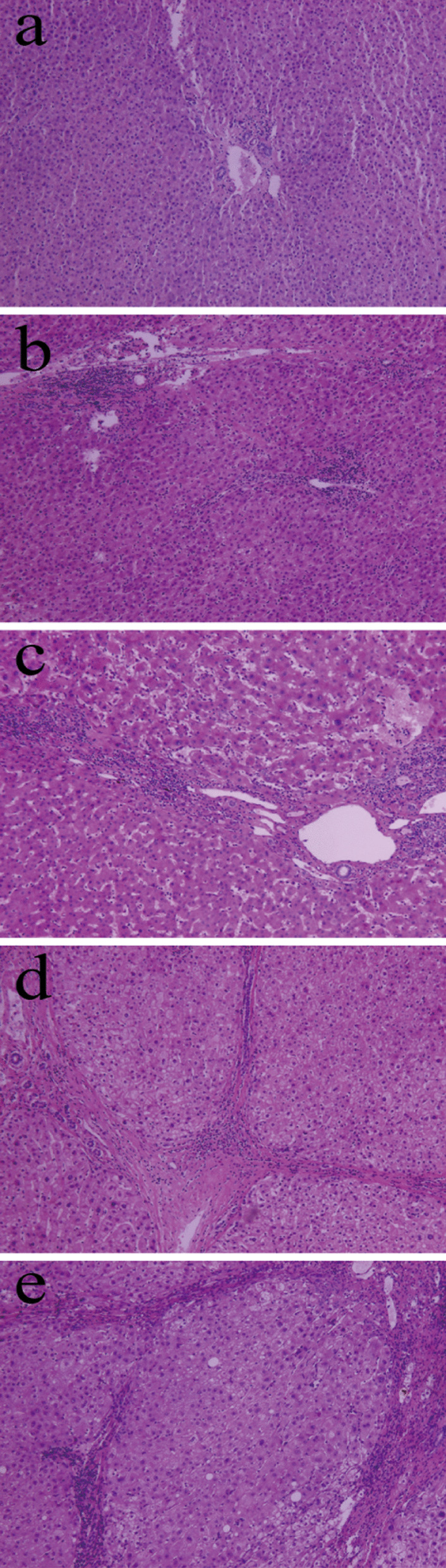
Representative micrographs showing postoperative pathological hepatic fibrosis staging in liver tissues after haematoxylin and eosin staining. Magnification, 40x. Examples illustrate fibrosis stages **a** F0, **b** F1, **c** F2, **d** F3, and **e** F4

Liver tissue samples were fixed in 10% buffered formalin, embedded in paraffin, and stained with haematoxylin and eosin (HE), Masson’s trichrome and reticular fiber. The assessment of hepatic fibrosis was performed according to the analysis of at least 1.5 cm of liver tissue containing at least five portal tracts. Two histologists independently confirmed the extent of hepatic fibrosis in each sample using the METAVIR scoring system [13]: F0, no fibrosis; F1, expansion of portal zones; F2, expansion of most portal zones and occasional bridging; F3, expansion of most portal zones and marked bridging and occasional modules; or F4, cirrhosis. The absence of clinically significant hepatic fibrosis was defined as hepatic fibrosis stages F0 or F1, while severe hepatic fibrosis was classified as F2 or F3, and cirrhosis as F4 (Fig. 3). Histologists were blinded to clinical data. Disagreements between histologists were resolved by discussion.

### Statistical analysis

Data from continuous variables were expressed as mean ± standard deviation, and differences in these variables were assessed for significance using Student’s *t* test, while differences in categorical variables were assessed using the chi-squared test. Independent risk factors for hepatic insufficiency were identified using logistic regression. Receiver operating characteristic (ROC) curves were plotted in order to determine threshold SRLVs for predicting hepatic insufficiency. Data analyses were performed using MedCalc analysis software 15.2.0 (Ostend, Belgium). All tests were 2-sided. Differences were considered significant when p˂ 0.05.

## Results

### Demographic and clinical characteristics of 85 HCC patients

A total of 85 patients were enrolled, including 70 (82.35%) men and 15 (17.65%) women (Table 1), and 56 of the 85 patients (65.88%) were HBsAg-positive. Sixty-five patients (76.47%) had a single tumour in the right half of the liver, while the remaining 20 (23.53%) had multiple tumours in the right half.

**Table 1:** Demographic and clinical characteristics of 85 Chinese HCC patients who underwent right hemi-hepatectomy

Parameter	Value
Sex	
Male	70 (82.35)
Female	15 (17.65)
Hepatitis B	
Positive	56 (65.88)
Negative	29 (34.12)
Tumour number	
Single	65 (76.47)
Multiple	20 (23.53)
*Preoperative parameters*	
Age (year)	44.71 ± 11.52
Platelet count (10^9^/L)	261.43 ± 85.68
Total bilirubin (μmol/l)	15.51 ± 11.87
Albumin (g/L)	39.05 ± 4.50
ALT (IU/L)	45.53 ± 26.09
AST (IU/L)	78.16 ± 62.08
Prothrombin time (s)	12.86 ± 1.21
Alpha-foetoprotein (μg/ml)	770.57 ± 1060.93
Body surface area (m^2^)	1.95 ± 0.16
Tumour size (cm)	11.37 ± 3.69
Remnant liver volume (ml)	552.18 ± 214.32
SRLV (ml/m^2^)	282.00 ± 96.20
Child–Pugh classification	
A	71 (83.53)
B	14 (16.47)
*Intraoperative parameters*	
Blood loss (ml)	716.67 ± 650.96
*Postoperative parameters*	
Tumour capsule	
Yes	75 (88.24)
No	10 (11.76)
Macrovascular invasion	
Yes	64 (75.29)
No	21 (24.71)
Hepatic fibrosis stage	
No significant fibrosis	31 (36.47)
Severe fibrosis	24 (28.24)
Cirrhosis	30 (35.29)
Hospital stay (days)	25.87 ± 6.56

Values are n (%) or mean ± SD

*ALP* alkaline phosphatase, *ALT* alanine transaminase, *AST* aspartate aminotransferase, *SRLV* standard remnant liver volume

### Risk factors for hepatic insufficiency

Among 85 HCC patients who underwent right hemi-hepatectomy, 30 (35.29%) developed postoperative hepatic insufficiency. Despite this, no patients died during the perioperative period, defined as from one week before surgery until one week after surgery. Three patients died in-hospital: one died of abdominal hemorrhage at two weeks after surgery, and two died of multiple organ failure at four weeks after surgery. All patients were transferred immediately to the intensive care unit for 1–2 days of short-term resuscitation. The following clinicodemographic variables differed significantly in frequency between patients who experienced insufficiency and those who did not (Table 2): height of the patient, PT, remnant liver volume, SRLV and hepatic fibrosis stage.

**Table 2:** Comparison of clinicopathologic characteristics between HCC patients who experienced hepatic insufficiency or not after right hemi-hepatectomy

Characteristic	Insufficiency (n = 30)	No insufficiency (n = 55)	χ^2^ (t test)	P
Sex				
Male	28	42	3.846	0.05
Female	2	13		
HBsAg				
Positive	20	36	0.013	0.91
Negative	10	19		
Tumour number				
Single	18	34	0.027	0.869
Multiple	12	21		
*Preoperative parameter*				
Age (year)	43.47 ± 9.09	45.38 ± 12.67	0.731	0.467
Platelet count (10^9^/L)	243.08 ± 95.26	271.44 ± 79.10	1.390	0.171
Total bilirubin (μmol/l)	16.51 ± 6.88	14.97 ± 13.89	− 0.686	0.495
Albumin (g/L)	39.62 ± 4.93	38.74 ± 4.26	− 0.824	0.414
ALT (IU/L)	49.27 ± 24.15	43.49 ± 27.08	− 1.009	0.317
AST (IU/L)	94.43 ± 84.22	69.29 ± 44.26	− 1.808	0.074
Prothrombin time (s)	13.33 ± 1.25	12.61 ± 1.11	− 2.612	0.012*
Alpha-foetoprotein (μg/ml)	821.73 ± 516.56	742.67 ± 1267.02	− 0.405	0.686
Body surface area (m^2^)	1.99 ± 0.15	1.93 ± 0.17	− 1.465	0.148
Tumour size (cm)	12.10 ± 2.79	10.98 ± 4.06	− 1.499	0.138
Remnant liver volume (ml)	489.89 ± 177.39	586.16 ± 226.31	2.164	0.034*
SRLV (ml/m^2^)	238.25 ± 62.14	305.85 ± 103.35	3.762	0.000**
Child–Pugh classification				
A	22	49	2.452	0.117
B	8	6		
*Intraoperative parameter*				
Blood loss (ml)	753.33 ± 623.52	687.27 ± 667.72	− 0.455	0.651
*Postoperative parameter*				
Tumour capsule				
Yes	25	49	0.174	0.676
No	5	6		
Macrovascular invasion				
Yes	26	39	2.679	0.102
No	4	16		
Hepatic fibrosis stage			6.863	0.032*
No significant fibrosis	7	24		
Severe fibrosis	7	17		
Cirrhosis	16	14		
Hospital stay (days)	27.37 ± 7.78	25.51 ± 5.39	− 1.785	0.069

Values are n (%) or mean ± SD, unless otherwise noted, *p < 0.05, **p < 0.01

*HBsAg* hepatitis B surface antigen, *ALT* alanine transaminase, *AST* aspartate aminotransferase, *BSA* body surface area, *SRLV* standard remnant liver volume

Binary logistic regression was performed to identify relationships of sex, height, PT, remnant liver volume, SRLV, or hepatic fibrosis stage with postoperative hepatic insufficiency using forward stepwise regression. The significance threshold for inclusion in the model was 0.05, and the threshold for removal was 0.1. This analysis identified SRLV and hepatic fibrosis stage (both p < 0.001) as independent risk factors for postoperative hepatic insufficiency after right hemi-hepatectomy for HCC (Table 3).

**Table 3:** Binary logistic regression analysis to identify factors related to hepatic insufficiency

Factor	B	Hazard Ratio (95% CI)	p
Sex (male)	2.008	7.451 (0.772–71.933)	0.083
Height	0.07	1.072 (0.933–1.232)	0.325
Prothrombin time	0.382	1.465 (0.837–2.561)	0.181
Remnant liver volume	0.006	1.006 (0.989–1.022)	0.514
SRLV	− 0.051	0.951 (0.914–0.989)	0.011*
*Hepatic fibrosis stage*			
Severe fibrosis	1.045	2.845 (0.471–17.174)	0.254
Cirrhosis	3.962	52.588 (5.547–498.512)	0.001**

*CI* confidence interval, *SRLV* standard remnant liver volume

*p < 0.05, **p < 0.01

### SRLV thresholds for hepatic insufficiency in HCC patients depending on hepatic fibrosis stage

**Fig. 4:**
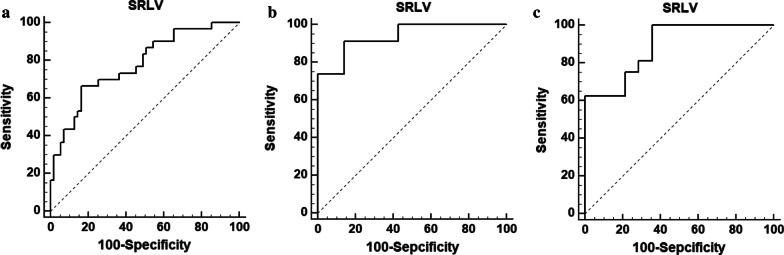
Receiver operating characteristic curve analysis of the ability of SRLV to predict postoperative insufficiency in **a** all HCC patients who underwent right hemi-hepatectomy, **b** the subgroup of patients with severe hepatic fibrosis, or **c** the subgroup of patients with cirrhosis

ROC analysis showed SRLV to have high sensitivity and specificity for predicting postoperative hepatic insufficiency in HCC patients. The SRLV threshold was 203.2 ml/m^2^ across all patients, which gave an area under the ROC curve (AUC) of 0.778, sensitivity of 66.67%, and specificity of 83.64%, (p < 0.0001, Fig. 4a). The SRLV threshold was 193.757 ml/m^2^ for patients with severe hepatic fibrosis (AUC 0.938, sensitivity 91.30%, specificity 85.71%, p < 0.0001, Fig. 4b) and 224.265 ml/m^2^ for patients with cirrhosis (AUC 0.888, sensitivity 100%, specificity 64.29%, p < 0.0001, Fig. 4c).

## Discussion

Liver resection is one of the main treatments for patients with HCC [14, 15], but postoperative hepatic insufficiency remains the leading cause of death in these patients. The International Study Group of Liver Surgery (ISGLS) criteria [12] and the 50-50 criteria [16] are widely used to assess the risk that a given patient will suffer postoperative insufficiency. Also widely used are the remnant liver volume [17, 18] and SRLV, which normalises the liver volume to body surface area in order to minimise individual differences. Currently there is no global standard for deciding the minimum remnant liver volume that should be ensured to avoid postoperative hepatic insufficiency. We present here threshold values for different subpopulations of HCC patients with cirrhosis or fibrosis after right hemi-hepatectomy, which tends to remove a larger proportion of total liver volume. Therefore the thresholds here may be more conservative and safer than limits proposed after analysis of left hemi-hepatectomy or a combination of right and left hemi-hepatectomy.

The present study found that SRLV was an independent risk factor for postoperative hepatic insufficiency, which is consistent with previous work showing that standard remnant liver volume and indocyanine green retention rate at 15 min were independent risk factors for postoperative insufficiency [7]. At the same time, our threshold SRLV of 203.2 ml/m^2^ is slightly lower than the threshold of 250 ml/m^2^ in a study of Japanese patients [8]. This difference may reflect the fact that we examined only patients who underwent right hemi-hepatectomy, while the other study aggregated patients who underwent the right or left procedure. Some studies have suggested that residual hepatocytes in right hemi-hepatectomy may release more cytokines than after the left procedure, which may facilitate liver regeneration [19–21]. The lower SRLV in our study may also reflect that only 66% of patients were positive for hepatitis B virus and only 35% had cirrhosis, both of which inhibit liver regeneration.

The threshold SRLV was higher in HCC patients with cirrhosis than in those with severe hepatic fibrosis. This may reflect the lower regenerative ability of residual liver tissue in patients with cirrhosis [22, 23]. In this study, we found that hepatic insufficiency is closely related to the severity of hepatic fibrosis, highlighting the need for non-invasive, preoperative techniques to assess the severity of hepatic fibrosis. One promising approach is PSR, which we have validated in HCC patients from the same area of China as in the present study [24].

The results from the present work should be interpreted with caution given that it involved only 30 patients who developed hepatic insufficiency after surgery, including 7 with severe hepatic fibrosis and 16 with cirrhosis. Therefore, our results should be validated and extended in larger studies, preferably ones that also analyse postoperative complications and grade of liver failure. It would also be interesting to validate our SRLV thresholds against baseline liver function based on active contrast uptake in preoperative hepatobiliary magnetic resonance imaging. Future work should analyse not only the absolute volume of the right half of the liver but also the proportion of the right half to the entire liver volume.

## Conclusions

Despite these limitations, the results of this small study suggest that SRLV and hepatic fibrosis stage are independent risk factors for postoperative hepatic insufficiency in HCC patients undergoing right hemi-hepatectomy. Our results also propose threshold SRLV values that, if validated in larger cohorts, may help guide HCC patient management.

## Data Availability

All data generated or analysed during this study are included in this published article.

## Abbreviations

SRLV: Standard remnant liver volume
HCC: Hepatocellular carcinoma
CT: Computer tomography
